# Supplementary material for: Assessment of emerging pretraining strategies in interpretable multimodal deep learning for cancer prognostication
Source: BioData Min. 2023 Jul 22;16:23. doi: 10.1186/s13040-023-00338-w (PMC10363299; doi:10.1186/s13040-023-00338-w)
Supplement: Supplementary file 1 — Additional file 1: S1. Dataset clinical characteristics. S2. Unimodal Prognostication Performance. TableS3. Unimodal boostrapped C-indices with 95% confidence intervals for Uni-Selfmodels. TableS4.Unimodal bootstrapped C-indices with 95% confidence intervals for Uni-Crossmodels. S5.Bar graph comparison of unimodal pretraining strategies performances bymodality. S6. C-indices of WSI GCNs pretrained using Uni-Cross-WSI with DNAm prediction as the crossmodal prediction target. S7. Unimodal boostrapped C-indices with 95% confidence intervals for clinicalmodels, both with and without stage. S8. 95% Confidence Interval UniTransferC-indices reported per subtype per method; note that finetuning to each subtypewas not performed at the unimodal level in this experiment. S9. Significance of incorporation of deeplearning hazards improving prognostic capability, in addition to using solelyclinical covariates and solely pTNM staging, using CoxPH models. S10. Most significant pathways identified byIntegrated Gradients on gene expression encoders of top performing multimodalmodels. S11. Most significant genes identified by Integrated Gradients on DNA methylation encoders of top performing multimodalmodels. S12. Enrichr pathway analysis of reportedsignificant genes from DNAm data, using BioPlanet pathways. S13. Visualized t-SNE embeddings of fusedfeatures extracted from Multi-Transfer prior to subtype finetuning. S14. Kaplan Meier curves for predicted risk, stratified by patients with thefollowing KIRC molecular subtypes: A) PBRM1 mutation, B) BAP1 mutation. S15. Relationship between the ability of the multimodal models to localize TILs andpredicted risk. S16. Kaplan Meier survival curves depictinginteraction between TIL identification by multimodal model and predicted risk:A) Indicators of high or low predicted risk, stratified by model localizationof TILs, B) Model localization of TILs, stratified by indicators of high or lowpredicted risk. S17. Integrated gradients scores for sig [file 13040_2023_338_MOESM1_ESM.docx]

**Supplementary Information**

**​​** S1: Dataset clinical characteristics

|  | BLCA | BRCA | HNSC | KIRC | LIHC | LUAD | PAAD | SKCM | Overall |
| --- | --- | --- | --- | --- | --- | --- | --- | --- | --- |
| n | 412 | 1098 | 528 | 537 | 377 | 585 | 185 | 470 | 4192 |
| age (mean (SD)) | 68.13 (10.61) | 58.63 (13.20) | 60.95 (11.93) | 60.61 (12.16) | 59.31 (13.45) | 65.28 (10.06) | 64.90 (11.06) | 58.26 (15.73) | 61.26 (12.98) |
| gender = male (%) | 304 (73.8) | 12 (1.1) | 386 (73.1) | 346 (64.4) | 255 (67.6) | 242 (46.4) | 102 (55.1) | 290 (61.7) | 1937 (46.9) |
| race (%) |  |  |  |  |  |  |  |  |  |
| american indian or alaska native | 0 (0.0) | 1 (0.1) | 2 (0.4) | 0 (0.0) | 2 (0.5) | 1 (0.2) | 0 (0.0) | 0 (0.0) | 6 (0.1) |
| asian | 44 (10.7) | 61 (5.6) | 11 (2.1) | 8 (1.5) | 161 (42.7) | 8 (1.5) | 11 (5.9) | 12 (2.6) | 316 (7.7) |
| black or african american | 23 (5.6) | 183 (16.7) | 48 (9.1) | 56 (10.4) | 17 (4.5) | 53 (10.2) | 7 (3.8) | 1 (0.2) | 388 (9.4) |
| not reported | 18 (4.4) | 95 (8.7) | 15 (2.8) | 7 (1.3) | 10 (2.7) | 67 (12.8) | 5 (2.7) | 10 (2.1) | 227 (5.5) |
| white | 327 (79.4) | 757 (69.0) | 452 (85.6) | 466 (86.8) | 187 (49.6) | 393 (75.3) | 162 (87.6) | 447 (95.1) | 3191 (77.3) |
| ethnicity (%) |  |  |  |  |  |  |  |  |  |
| hispanic or latino | 9 (2.2) | 39 (3.6) | 26 (4.9) | 26 (4.8) | 18 (4.8) | 7 (1.3) | 5 (2.7) | 11 (2.3) | 141 (3.4) |
| not hispanic or latino | 371 (90.0) | 884 (80.6) | 465 (88.1) | 359 (66.9) | 340 (90.2) | 389 (74.5) | 137 (74.1) | 446 (94.9) | 3391 (82.1) |
| not reported | 32 (7.8) | 174 (15.9) | 37 (7.0) | 152 (28.3) | 19 (5.0) | 126 (24.1) | 43 (23.2) | 13 (2.8) | 596 (14.4) |
| ajcc_pathologic_stage (%) |  |  |  |  |  |  |  |  |  |
| Not Reported | 0 (0.0) | 0 (0.0) | 0 (0.0) | 0 (0.0) | 0 (0.0) | 0 (0.0) | 0 (0.0) | 14 (3.2) | 14 (0.4) |
| Stage 0 | 0 (0.0) | 0 (0.0) | 0 (0.0) | 0 (0.0) | 0 (0.0) | 0 (0.0) | 0 (0.0) | 7 (1.6) | 7 (0.2) |
| Stage I | 2 (0.5) | 183 (16.9) | 27 (6.0) | 269 (50.4) | 175 (49.6) | 279 (54.3) | 21 (11.5) | 77 (17.8) | 1033 (26.1) |
| Stage II | 131 (32.0) | 621 (57.2) | 74 (16.3) | 57 (10.7) | 87 (24.6) | 124 (24.1) | 152 (83.5) | 140 (32.4) | 1386 (35.0) |
| Stage III | 141 (34.4) | 249 (22.9) | 82 (18.1) | 125 (23.4) | 86 (24.4) | 85 (16.5) | 4 (2.2) | 171 (39.6) | 943 (23.8) |
| Stage IV | 136 (33.2) | 20 (1.8) | 270 (59.6) | 83 (15.5) | 5 (1.4) | 26 (5.1) | 5 (2.7) | 23 (5.3) | 568 (14.3) |
| Stage X | 0 (0.0) | 13 (1.2) | 0 (0.0) | 0 (0.0) | 0 (0.0) | 0 (0.0) | 0 (0.0) | 0 (0.0) | 13 (0.3) |
| days_to_death (mean (SD)) | 550.82 (533.62) | 1584.62 (1312.07) | 738.26 (923.07) | 926.72 (741.28) | 669.14 (695.04) | 790.41 (700.86) | 459.27 (362.68) | 1782.98 (1888.91) | 980.13 (1159.26) |
| ajcc_pathologic_t (%) |  |  |  |  |  |  |  |  |  |
| T0 | 1 (0.3) | 0 (0.0) | 1 (0.2) | 0 (0.0) | 0 (0.0) | 0 (0.0) | 0 (0.0) | 23 (5.2) | 25 (0.6) |
| T1 | 3 (0.8) | 281 (25.6) | 49 (9.7) | 275 (51.2) | 185 (49.3) | 172 (33.0) | 7 (3.8) | 42 (9.5) | 1014 (25.1) |
| T2 | 120 (31.6) | 635 (57.9) | 140 (27.7) | 69 (12.8) | 95 (25.3) | 281 (53.8) | 24 (13.0) | 78 (17.7) | 1442 (35.7) |
| T3 | 196 (51.6) | 138 (12.6) | 101 (20.0) | 182 (33.9) | 81 (21.6) | 47 (9.0) | 148 (80.4) | 90 (20.4) | 983 (24.3) |
| T4 | 59 (15.5) | 40 (3.6) | 175 (34.7) | 11 (2.0) | 13 (3.5) | 19 (3.6) | 4 (2.2) | 153 (34.7) | 474 (11.7) |
| Tis | 0 (0.0) | 0 (0.0) | 0 (0.0) | 0 (0.0) | 0 (0.0) | 0 (0.0) | 0 (0.0) | 8 (1.8) | 8 (0.2) |
| TX | 1 (0.3) | 3 (0.3) | 39 (7.7) | 0 (0.0) | 1 (0.3) | 3 (0.6) | 1 (0.5) | 47 (10.7) | 95 (2.4) |
| ajcc_pathologic_n (%) |  |  |  |  |  |  |  |  |  |
| N0 | 239 (58.9) | 516 (47.0) | 180 (35.8) | 240 (44.7) | 257 (68.4) | 335 (64.3) | 50 (27.2) | 235 (52.3) | 2052 (50.4) |
| N1 | 47 (11.6) | 364 (33.2) | 68 (13.5) | 17 (3.2) | 4 (1.1) | 98 (18.8) | 130 (70.7) | 74 (16.5) | 802 (19.7) |
| N2 | 76 (18.7) | 120 (10.9) | 172 (34.2) | 0 (0.0) | 0 (0.0) | 75 (14.4) | 0 (0.0) | 49 (10.9) | 492 (12.1) |
| N3 | 8 (2.0) | 77 (7.0) | 8 (1.6) | 0 (0.0) | 0 (0.0) | 2 (0.4) | 0 (0.0) | 55 (12.2) | 150 (3.7) |
| NX | 36 (8.9) | 20 (1.8) | 75 (14.9) | 280 (52.1) | 115 (30.6) | 11 (2.1) | 4 (2.2) | 36 (8.0) | 577 (14.2) |
| ajcc_pathologic_m (%) |  |  |  |  |  |  |  |  |  |
| M0 | 196 (47.9) | 912 (83.1) | 191 (74.3) | 426 (79.6) | 272 (72.1) | 353 (68.1) | 85 (45.9) | 418 (94.6) | 2853 (74.7) |
| M1 | 11 (2.7) | 22 (2.0) | 1 (0.4) | 79 (14.8) | 4 (1.1) | 25 (4.8) | 5 (2.7) | 24 (5.4) | 171 (4.5) |
| MX | 202 (49.4) | 163 (14.9) | 65 (25.3) | 30 (5.6) | 101 (26.8) | 140 (27.0) | 95 (51.4) | 0 (0.0) | 796 (20.8) |

**S2 – Unimodal Prognostication Performance**

Unimodal models were trained to predict survival using standard self-supervised pretraining procedure (Uni-Self), crossmodal pretraining (Uni-Cross), and transfer learning (Uni-Transfer). Separately, clinical models were trained to predict survival using age, race, sex, and both with/without pathological stage.

In **Appendix Table S3** we report the 95% confidence interval bootstrapped C-indices for each subtype for the Uni-Self models, and we report the same statistic for the Uni-Cross models in **Appendix Table S4**. These mechanisms are compared in **Appendix Figure S5** as well. Results for the Uni-Cross-WSI model, when DNAm was used as the crossmodal target, are reported in **Appendix Table S6**. Performance of baseline clinical models (trained using age, race, sex, with/without stage) is presented in in **Appendix Table S7**. C-index performance for Uni-Transfer models is reported in **Appendix Table S8** (not finetuned on each subtype).

WSI GCNs outperform DNAm and gene expression unimodal models on 5/8 subtypes with the standard Uni-Self models, while gene expression becomes the top-performing modality on more than half of subtypes when crossmodal pretraining is implemented with the Uni-Cross models. All Uni-Cross gene expression models outperformed the corresponding Uni-Self models (6.62% average increase). Uni-Cross DNAm models brought performance improvements on 5/8 subtypes compared to the corresponding Uni-Self models (increase average 2.89%). Uni-Cross WSI GCNs also brought improvement on 5/8 subtypes compared to the Uni-Self setup (increase average 2.07%). Every subtype benefitted from crossmodal pretraining for at least one modality. Top performing Uni-Self or Uni-Pre models outperformed top-performing clinical models which did not include stage for all subtypes (increase average 34.05%), and outperformed clinical models which did include stage (increase average 12.44%), for all subtypes besides BLCA.

**Table S3:** Unimodal boostrapped C-indices with 95% confidence intervals for Uni-Self models

| **Subtype** | **DNAm** | **Gene Expression** | **WSI GCN** |
| --- | --- | --- | --- |
| BLCA | 0.633 ± 0.002 | **0.689 ± 0.002** | 0.564 ± 0.003 |
| BRCA | **0.711**  ± **0.002** | 0.547 ± 0.002 | 0.558 ± 0.003 |
| HNSC | 0.600 ± 0.002 | 0.608 ± 0.002 | **0.680 ± 0.002** |
| KIRC | 0.699 ± 0.003 | 0.691 ± 0.005 | **0.777 ± 0.004** |
| LIHC | 0.562 ± 0.003 | 0.569 ± 0.002 | **0.722 ± 0.003** |
| LUAD | 0.645 ± 0.003 | 0.601 ± 0.002 | **0.663 ± 0.003** |
| PAAD | 0.603 ± 0.004 | 0.585 ± 0.004 | **0.616 ± 0.004** |
| SKCM | 0.588 ± 0.003 | **0.632 ± 0.003** | 0.623 ± 0.002 |

**Table S4:** Unimodal bootstrapped C-indices with 95% confidence intervals for Uni-Cross models

| **Subtype** | **DNAm** | **Gene Expression** | **WSI GCN** |
| --- | --- | --- | --- |
| BLCA | 0.619 ± 0.003 | **0.692 ± 0.002** | 0.571 ± 0.002 |
| BRCA | 0.552 ± 0.003 | **0.624 ± 0.002** | 0.573 ± 0.003 |
| HNSC | 0.623 ± 0.002 | **0.648 ± 0.002** | 0.606 ± 0.003 |
| KIRC | 0.688 ± 0.002 | 0.713 ± 0.002 | **0.794 ± 0.004** |
| LIHC | 0.618 ± 0.003 | 0.655 ± 0.003 | **0.746 ± 0.002** |
| LUAD | 0.646 ± 0.002 | 0.631± 0.004 | **0.669 ± 0.003** |
| PAAD | 0.604 ± 0.004 | **0.628 ± 0.004** | 0.575 ± 0.004 |
| SKCM | 0.590 ± 0.003 | **0.640 ± 0.003** | 0.559 ± 0.002 |


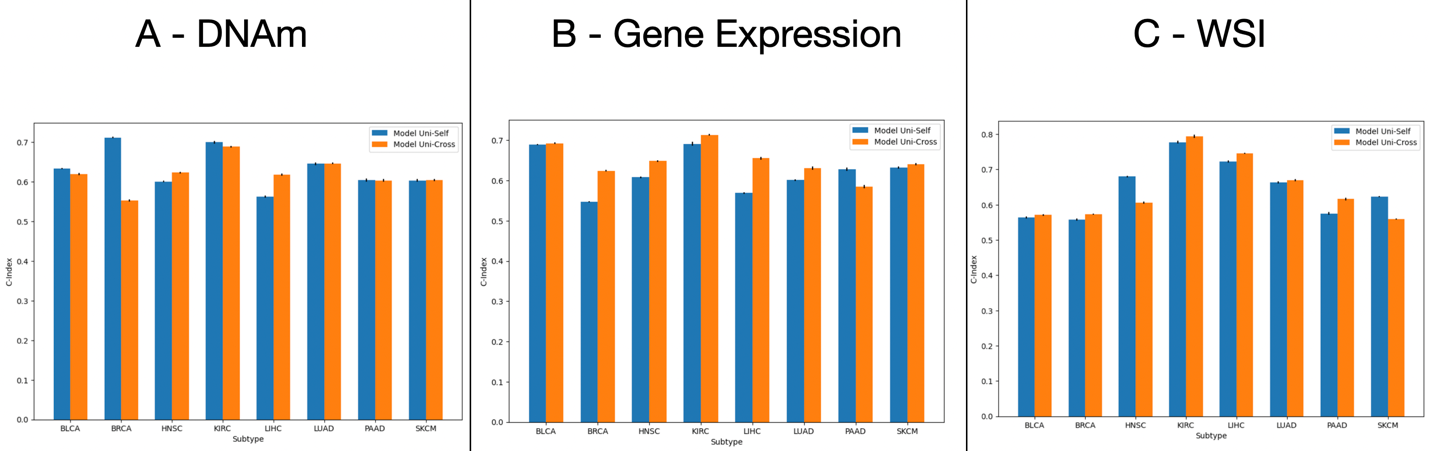


S5**:** Bar graph comparison of unimodal pretraining strategies performances by modality

S6: C-indices of WSI GCNs pretrained using Uni-Cross-WSI with DNAm prediction as the crossmodal prediction target.

| **Subtype** | **C-Index** |
| --- | --- |
| BLCA | 0.551 ± 0.004 |
| BRCA | 0.600 ± 0.003 |
| HNSC | 0.601 ± 0.002 |
| KIRC | 0.770 ± 0.002 |
| LIHC | 0.741 ± 0.003 |
| LUAD | 0.676 ± 0.003 |
| PAAD | 0.453 ± 0.002 |
| SKCM | 0.562 ± 0.001 |

S7**:** Unimodal boostrapped C-indices with 95% confidence intervals for clinical models, both with and without stage

| **Subtype** | **RF- No Stage** | **RF-Stage** | **CoxPH- No Stage** | **CoxPH-Stage** |
| --- | --- | --- | --- | --- |
| BLCA | 0.585 ± 0.009 | **0.699 ± 0.006** | 0.590 ± 0.009 | 0.637 ± 0.005 |
| BRCA | 0.511 ± 0.010 | 0.589 ± 0.010 | 0.504 ± 0.011 | **0.590 ± 0.009** |
| HNSC | 0.542 ± 0.006 | 0.606 ± 0.007 | 0.547 ± 0.007 | **0.609 ± 0.006** |
| KIRC | 0.576 ± 0.011 | 0.784 ± 0.008 | 0.585 ± 0.013 | **0.787 ± 0.008** |
| LIHC | 0.525 ± 0.008 | 0.663 ± 0.008 | 0.531 ± 0.008 | **0.660 ± 0.008** |
| LUAD | 0.465 ± 0.012 | 0.621 ± 0.012 | 0.466 ± 0.012 | **0.622 ± 0.011** |
| PAAD | **0.516 ± 0.007** | 0.497 ± 0.008 | 0.512 ± 0.008 | 0.489 ± 0.008 |
| SKCM | 0.464 ± 0.007 | 0.562 ± 0.012 | 0.466 ± 0.007 | **0.573 ± 0.012** |

S8: 95% Confidence Interval UniTransfer C-indices reported per subtype per method; note that finetuning to each subtype was not performed at the unimodal level in this experiment

| **Subtype** | **Uni-Transfer-Omics (Gene Expression)** | **Uni-Transfer-Omics (DNAM)** | **Uni-Transfer-WSI** |
| --- | --- | --- | --- |
| **BLCA** | 0.674 ± 0.002 | 0.660 ± 0.002 | 0.582 ± 0.003 |
| **BRCA** | 0.742 ± 0.003 | 0.623 ± 0.002 | 0.752 ± 0.002 |
| **HNSC** | 0.626 ± 0.002 | 0.554 ± 0.002 | 0.445 ± 0.002 |
| **KIRC** | 0.552 ± 0.003 | 0.324 ± 0.002 | 0.719 ± 0.003 |
| **LIHC** | 0.582 ± 0.004 | 0.573 ± 0.003 | 0.626 ± 0.004 |
| **LUAD** | 0.610 ± 0.004 | 0.637 ± 0.002 | 0.657 ± 0.003 |
| **PAAD** | 0.590 ± 0.004 | 0.542 ± 0.005 | 0.505 ± 0.004 |
| **SKCM** | 0.577 ± 0.002 | 0.545 ± 0.003 | 0.434 ± 0.002 |

S9: Significance of incorporation of deep learning hazards improving prognostic capability, in addition to using solely clinical covariates and solely pTNM staging, using CoxPH models

| **Subtype** | **H1: Hazard+Stage>Hazard** | **H2: Hazard+Stage>Stage** |
| --- | --- | --- |
| BLCA | 0.057 | 0.120 |
| BRCA | 0.187 | 0.247 |
| HNSC | 0.071 | 0.085 |
| KIRC | 0.034 | 0.111 |
| LIHC | 0.148 | 0.119 |
| LUAD | 0.132 | 0.123 |
| PAAD | 0.168 | 0.319 |
| SKCM | 0.030 | 0.099 |

S10: Most significant pathways identified by Integrated Gradients on gene expression encoders of top performing multimodal models

| **BLCA** | **BRCA** | **HNSC** | **KIRC** | **LIHC** | **LUAD** | **PAAD** | **SKCM** |
| --- | --- | --- | --- | --- | --- | --- | --- |
| positive regulation of mitotic nuclear division (GO:0045840) | regulation of cellular localization (GO:0060341) | defense response to Gram-negative bacterium (GO:0050829) | positive regulation of actin nucleation (GO:0051127) | positive regulation of vasoconstriction (GO:0045907) | killing by host of symbiont cells (GO:0051873) | killing by host of symbiont cells (GO:0051873) | skeletal system development (GO:0001501) |
| regulation of smooth muscle cell proliferation (GO:0048660) | supramolecular fiber organization (GO:0097435) | mesoderm formation (GO:0001707) | regulation of mitotic nuclear division (GO:0007088) | negative regulation of Wnt signaling pathway (GO:0030178) | antibacterial humoral response (GO:0019731) | regulation of cell-matrix adhesion (GO:0001952) | killing by host of symbiont cells (GO:0051873) |
| kidney development (GO:0001822) | mammary gland development (GO:0030879) | intermediate filament bundle assembly (GO:0045110) | translational elongation (GO:0006414) | negative regulation of production of molecular mediator of immune response (GO:0002701) | antimicrobial humoral immune response mediated by antimicrobial peptide (GO:0061844) | receptor clustering (GO:0043113) | positive regulation of non-canonical Wnt signaling pathway (GO:2000052) |
| regulation of type 2 immune response (GO:0002828) | Fc receptor mediated stimulatory signaling pathway (GO:0002431) | regulation of leukocyte activation (GO:0002694) | receptor catabolic process (GO:0032801) | heme biosynthetic process (GO:0006783) | organic hydroxy compound transport (GO:0015850) | regulation of pri-miRNA transcription by RNA polymerase II (GO:1902893) | epidermis development (GO:0008544) |
| development of primary male sexual characteristics (GO:0046546) | regulation of macrophage activation (GO:0043030) | chemical synaptic transmission (GO:0007268) | negative regulation of actin filament polymerization (GO:0030837) | cellular response to starvation (GO:0009267) | positive regulation of cellular process (GO:0048522) | muscle filament sliding (GO:0030049) | positive regulation of cell differentiation (GO:0045597) |
| organic hydroxy compound transport (GO:0015850) | regulation of heterotypic cell-cell adhesion (GO:0034114) | response to interferon-gamma (GO:0034341) | positive regulation of NIK/NF-kappaB signaling (GO:1901224) | iron ion homeostasis (GO:0055072) | skeletal system development (GO:0001501) | hydrogen peroxide biosynthetic process (GO:0050665) | positive regulation of cellular process (GO:0048522) |
| terpenoid metabolic process (GO:0006721) | regulation of cysteine-type endopeptidase activity (GO:2000116) | hemidesmosome assembly (GO:0031581) | positive regulation of neuron death (GO:1901216) | very-low-density lipoprotein particle remodeling (GO:0034372) | cellular metal ion homeostasis (GO:0006875) | Fc receptor signaling pathway (GO:0038093) | adenylate cyclase-modulating G protein-coupled receptor signaling pathway (GO:0007188) |
| sensory perception of sound (GO:0007605) | alpha-linolenic acid metabolic process (GO:0036109) | establishment of skin barrier (GO:0061436) | regulation of phagocytosis, engulfment (GO:0060099) | zymogen activation (GO:0031638) | cellular response to fatty acid (GO:0071398) | positive regulation of cell population proliferation (GO:0008284) | antimicrobial humoral immune response mediated by antimicrobial peptide (GO:0061844) |
| organic acid transport (GO:0015849) | negative regulation of cell motility (GO:2000146) | intermediate filament organization (GO:0045109) | autophagy of peroxisome (GO:0030242) | protein processing (GO:0016485) | C21-steroid hormone metabolic process (GO:0008207) | defense response to Gram-positive bacterium (GO:0050830) | regulation of focal adhesion assembly (GO:0051893) |
| organic heteropentacyclic compound metabolic process (GO:1901376) | innate immune response in mucosa (GO:0002227) | regulation of water loss via skin (GO:0033561) | maintenance of blood-brain barrier (GO:0035633) | epithelial cell differentiation (GO:0030855) | positive regulation of granulocyte chemotaxis (GO:0071624) | positive regulation of coagulation (GO:0050820) | skin development (GO:0043588) |

S11: Most significant genes identified by Integrated Gradients on DNA methylation encoders of top performing multimodal models

| BLCA | BRCA | HNSC | KIRC | LIHC | LUAD | PAAD | SKCM |
| --- | --- | --- | --- | --- | --- | --- | --- |
| GSTA3 | IMPDH2 | CLPS | PDE11A | EEF1D | DAZAP1 | SS18L1 | IGFL2 |
| RAX | TOR2A | BCAP31 | DOK7 | JRK | LOC284788 | VDAC1 | RP2 |
| LOC100190940 | FPR2 | LOC340074 | CDH15 | OTOP2 | RAC2 | HCFC1R1 | HRCT1 |
| ZNF214 | SSBP3 | MDH1B | CLDN16 | EP400 | ARHGEF11 | VGLL1 | KDM2B |
| TEPP | CLEC17A | CNTFR | DAXX | VASN | LILRP2 | UXT | PCDH15 |
| PLOD2 | PPP1CC | ABL2 | KNDC1 | KIAA0114 | ZCRB1 | LILRP2 | GOLGA8A |
| GNAS | GNG7 | TMEM102 | FRMD4A | IDS | LOC729156 | MAGOH | PI4K2A |
| EFCAB6 | INPP5D | NCAM1 | VANGL2 | NR2E1 | ICOS | SNORD62B | ZCRB1 |
| GATA2 | SCRN3 | THRA | HOXC6 | ZFP41 | MAGOH | ICOS | MAGOH |
| ZNF354C | CCNA2 | PDK2 | PRDM16 | NSUN4 | CCNA2 | CCNA2 | SNORD62B |

S12: Enrichr pathway analysis of reported significant genes from DNAm data, using BioPlanet pathways

| **Subtype** | **Pathway** | **Adj. P-Value** |
| --- | --- | --- |
| **BLCA** | Rapid glucocorticoid receptor pathway | 0.004492 |
|  | Attenuation of GPCR signaling | 0.005488 |
|  | Beta-arrestins in GPCR desensitization | 0.005985 |
|  | LPA4-mediated signaling events | 0.007973 |
|  | Beta-arrestin-dependent recruitment of Src kinases in GPCR signaling | 0.008469 |
|  | Ion channel function in vascular endothelium | 0.008469 |
|  | Corticosteroids and cardioprotection | 0.008966 |
|  | Serotonin receptor 4/6/7 and NR3C signaling | 0.008966 |
|  | PKA activation in glucagon signaling | 0.008966 |
|  | GATA3-mediated activation of Th2 cytokine expression | 0.009957 |
| **BRCA** | G2 phase pathway | 0.002498 |
|  | Formyl peptide interaction with formyl peptide receptors | 0.003994 |
|  | Purine ribonucleoside monophosphate biosynthesis | 0.005488 |
|  | Platelet endothelial cell adhesion molecule 1 (PECAM1) interactions | 0.005985 |
|  | Hormone-sensitive lipase (HSL)-mediated triacylglycerol hydrolysis | 0.005985 |
|  | PI3K class IB pathway | 0.006482 |
|  | Cyclin A/B1-associated events during G2/M transition | 0.007476 |
|  | Presynaptic function of kainate receptors | 0.01045 |
|  | T cell receptor downstream signaling | 0.01095 |
|  | Signaling events mediated by PRL | 0.01144 |
| **HNSC** | Vitamin A uptake in enterocytes | 0.003495 |
|  | Abl role in Robo-Slit signaling | 0.004492 |
|  | Pyruvate dehydrogenase (PDH) complex regulation | 0.005985 |
|  | MAP kinase inactivation of SMRT corepressor | 0.006979 |
|  | Apoptotic cleavage of cellular proteins | 0.008469 |
|  | Synaptic proteins at the synaptic junction | 0.008469 |
|  | PTEN-dependent cell cycle arrest and apoptosis | 0.009462 |
|  | Skeletal muscle hypertrophy is regulated via AKT/mTOR pathway | 0.009957 |
|  | Y branching of actin filaments | 0.01045 |
|  | RXR/VDR pathway | 0.01293 |
| **KIRC** | Stress induction of HSP regulation | 0.007476 |
|  | Transcriptional activity regulation by PML | 0.008966 |
|  | Cell junction organization | 0.0007674 |
|  | Nitric oxide stimulation of guanylate cyclase | 0.01392 |
|  | Adherens junction actin cytoskeletal organization | 0.01441 |
|  | CDO in myogenesis | 0.01441 |
|  | Tight junction interactions | 0.01490 |
|  | Cell-cell communication | 0.001796 |
|  | Cell adhesion molecules (CAMs) | 0.001907 |
|  | FAS pathway and stress induction of heat shock protein regulation | 0.01933 |
| **LIHC** | Chondroitin sulfate/dermatan sulfate degradation | 0.006482 |
|  | Glycosaminoglycan degradation | 0.009462 |
|  | Heparan sulfate/heparin glycosaminoglycan (HS-GAG) degradation | 0.009957 |
|  | Nuclear receptors | 0.01884 |
|  | Chondroitin sulfate/dermatan sulfate metabolism | 0.02375 |
|  | Translation factors | 0.02473 |
|  | Nuclear receptor transcription pathway | 0.02521 |
|  | Heparan sulfate/heparin glycosaminoglycan (HS-GAG) metabolism | 0.02570 |
|  | Glycosaminoglycan metabolism | 0.05367 |
|  | Lysosome | 0.05889 |
| **LUAD** | G2 phase pathway | 0.002498 |
|  | Sema4D in semaphorin signaling | 0.00009070 |
|  | Cyclin A/B1-associated events during G2/M transition | 0.007476 |
|  | Semaphorin interactions | 0.0004745 |
|  | STAT3 pathway | 0.009462 |
|  | T cell activation co-stimulatory signal | 0.01045 |
|  | G alpha (12/13) signaling events | 0.0006453 |
|  | Signaling events mediated by PRL | 0.01144 |
|  | Rho cell motility signaling pathway | 0.01144 |
|  | G0 and early G1 pathway | 0.01243 |
| **PAAD** | G2 phase pathway | 0.002498 |
|  | Cyclin A/B1-associated events during G2/M transition | 0.007476 |
|  | E2F transcription factor network | 0.0005962 |
|  | T cell activation co-stimulatory signal | 0.01045 |
|  | Signaling events mediated by PRL | 0.01144 |
|  | G0 and early G1 pathway | 0.01243 |
|  | Interleukin-2/STAT5 pathway | 0.01490 |
|  | Primary immunodeficiency | 0.01737 |
|  | FRA pathway | 0.01835 |
|  | FOXM1 transcription factor network | 0.02032 |
| **SKCM** | PIP biosynthesis at the early endosome membrane | 0.006482 |
|  | PIP biosynthesis at the Golgi membrane | 0.008469 |
|  | PIP biosynthesis at the plasma membrane | 0.01638 |
|  | Cleavage of growing transcript in the termination region | 0.02130 |
|  | Phosphatidylinositol metabolism | 0.02473 |
|  | Transport of mature transcript to cytoplasm | 0.02717 |
|  | Inositol phosphate metabolism | 0.02814 |
|  | Messenger RNA splicing: major pathway | 0.03349 |
|  | Phosphatidylinositol signaling system | 0.03833 |
|  | RNA polymerase II transcription | 0.04938 |


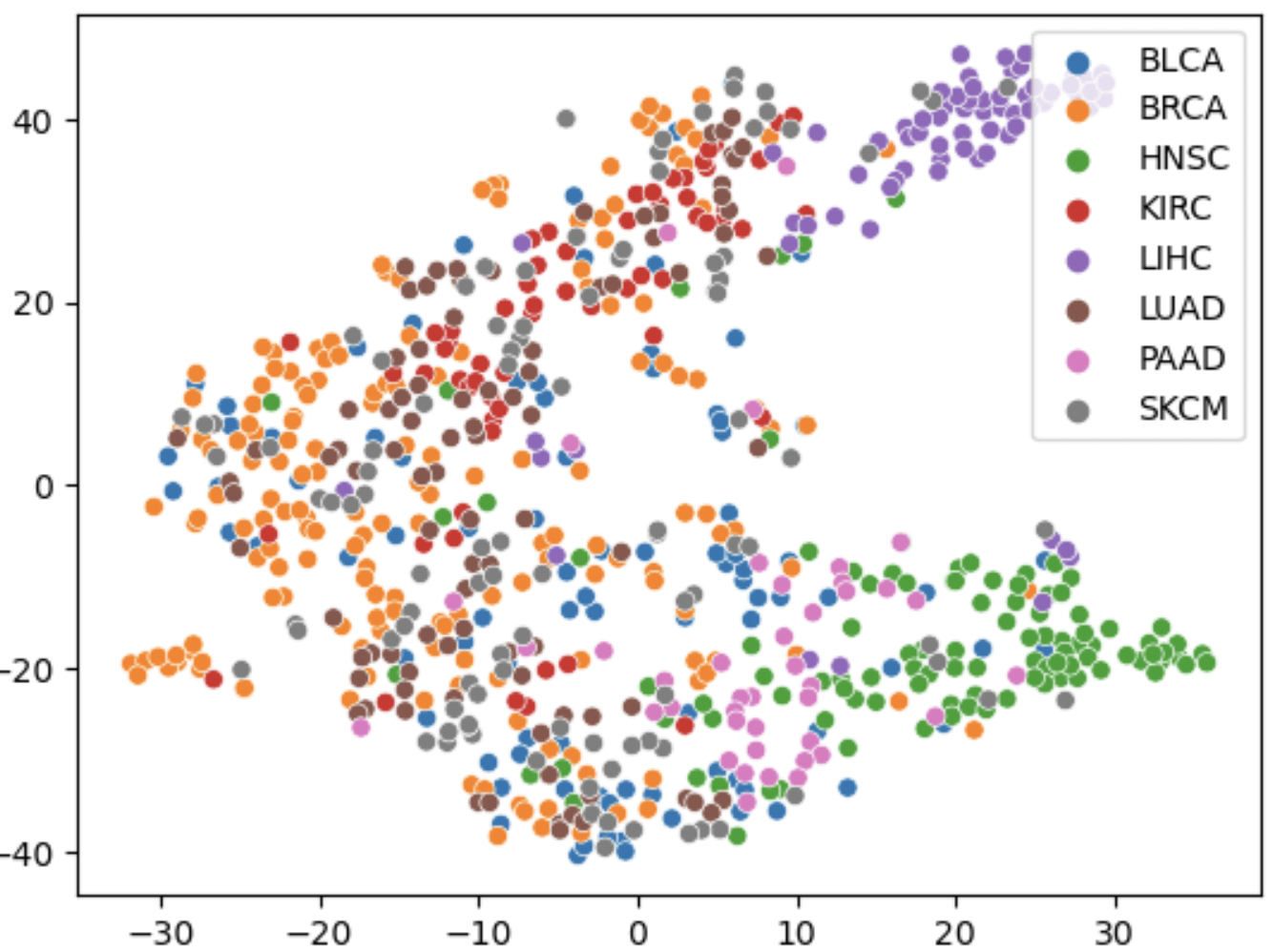


S13: Visualized t-SNE embeddings of fused features extracted from Multi-Transfer prior to subtype finetuning


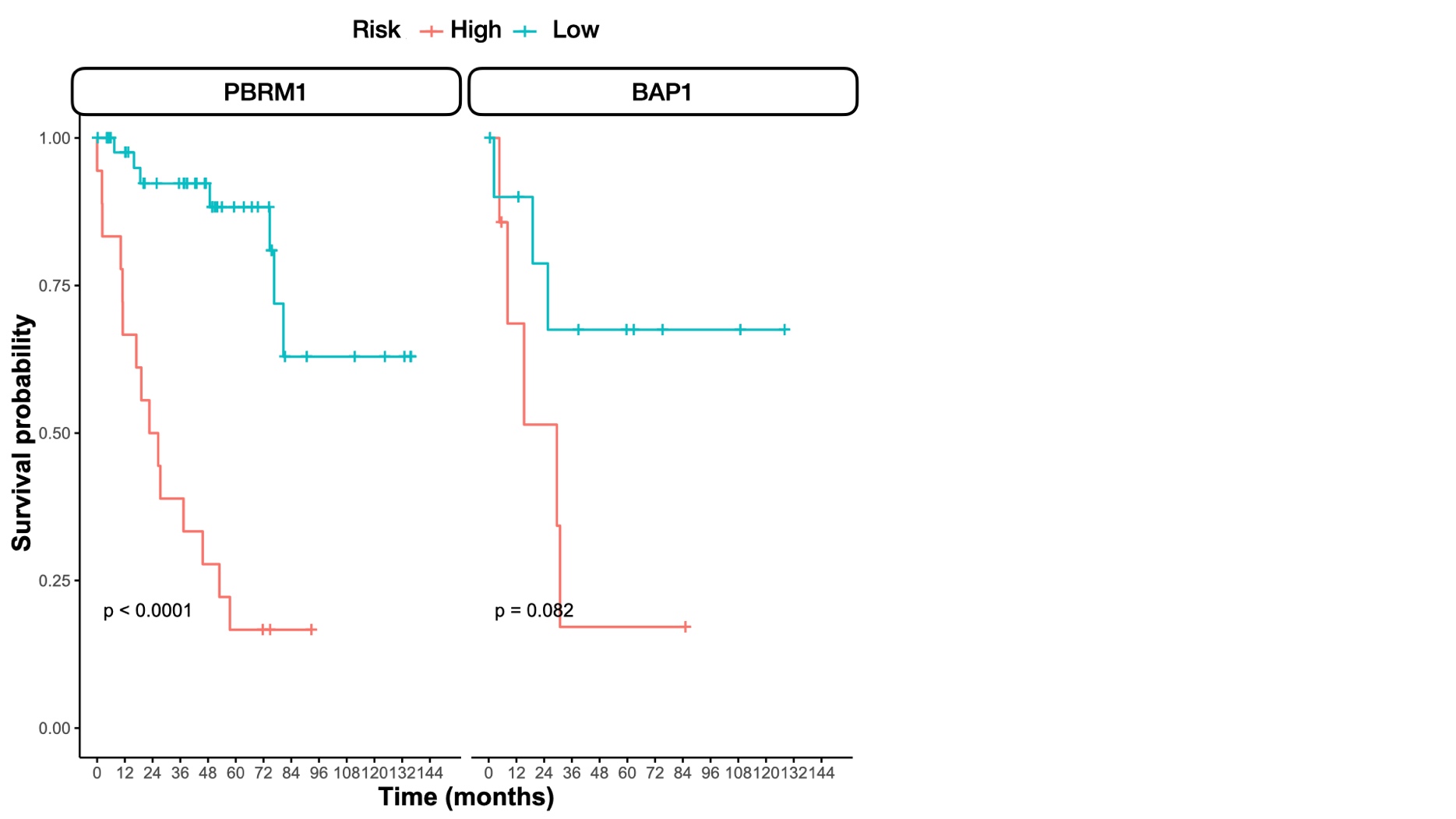


S14: Kaplan Meier curves for predicted risk, stratified by patients with the following KIRC molecular subtypes: A) PBRM1 mutation, B) BAP1 mutation

S15**:** Relationship between the ability of the multimodal models to localize TILs and predicted risk

| **Subtype** | **Odds Ratio** | **P-Value** |
| --- | --- | --- |
| BLCA | 0.44 | 0.0029 |
| BRCA | 0.56 | 0.1208 |
| LUAD | 1.76 | 0.0132 |
| PAAD | 0.33 | 0.0087 |
| SKCM | 2.11 | 0.0187 |


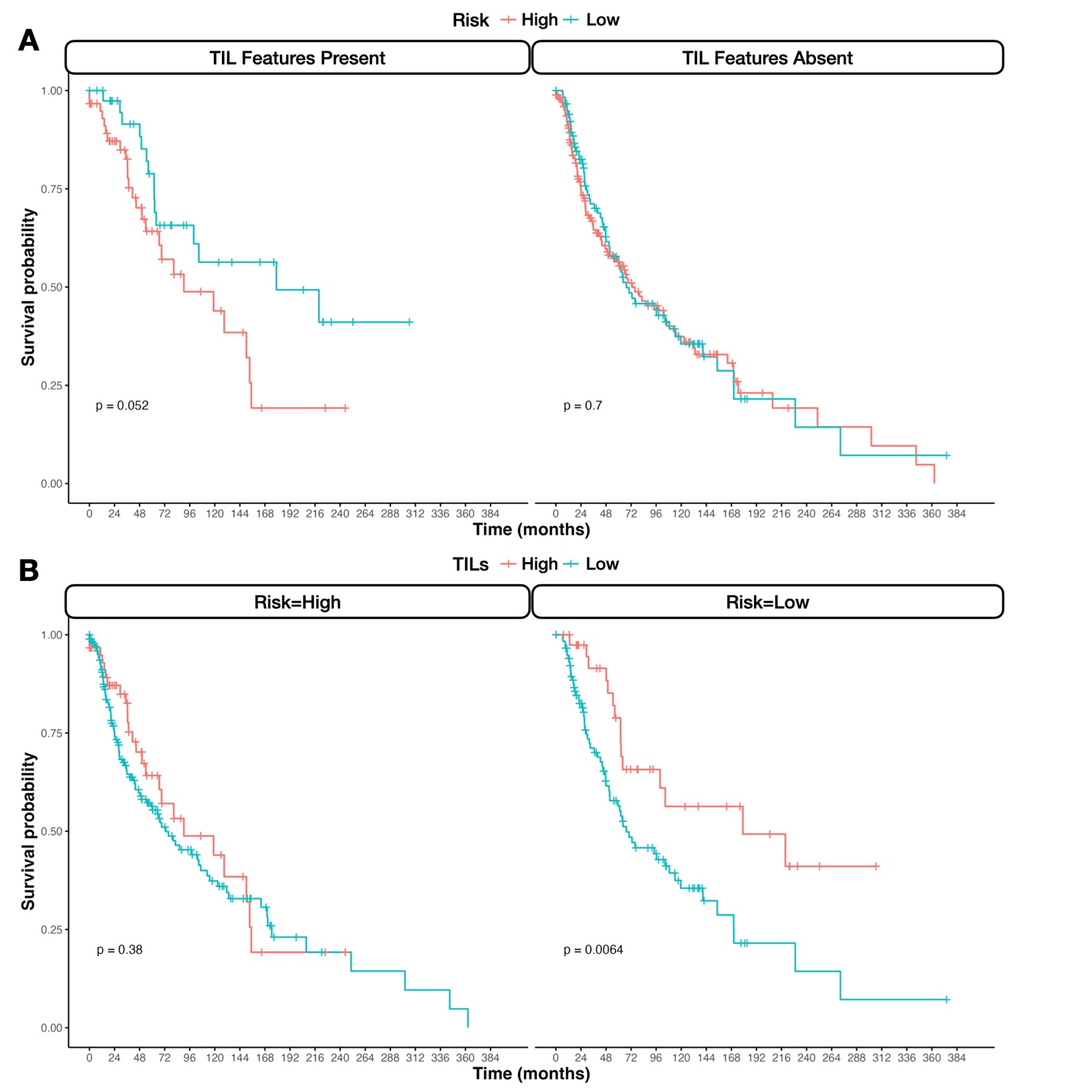


S16: Kaplan Meier survival curves depicting interaction between TIL identification by multimodal model and predicted risk: A) Indicators of high or low predicted risk, stratified by model localization of TILs, B) Model localization of TILs, stratified by indicators of high or low predicted risk

S17: Integrated gradients scores for significant genes derived from top-performing multimodal model DNAm networks. **See excel file, S17_DNAm_IG.xlsx**


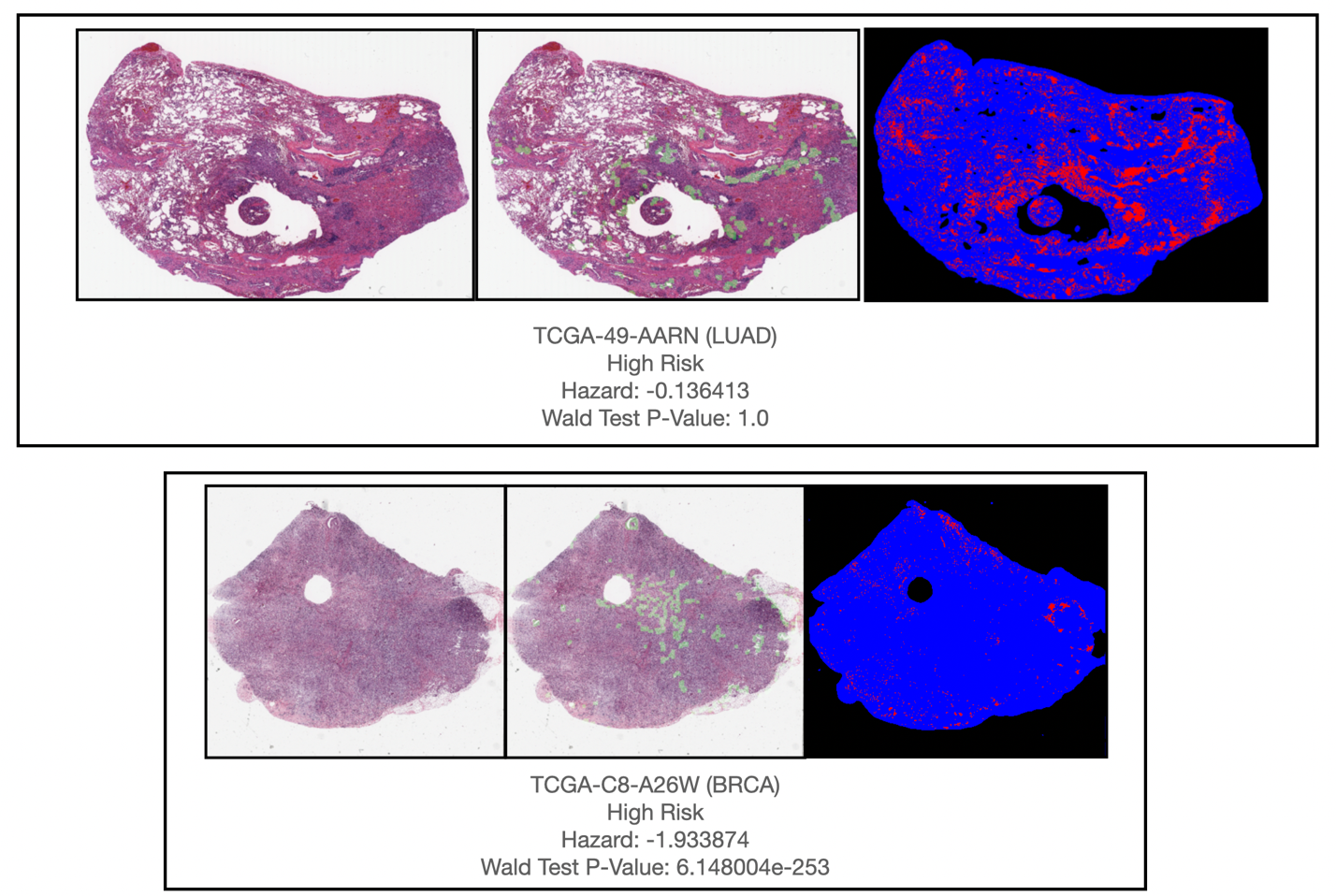


S18: Additional interpretation of WSI GCNs from top performing multimodal models

S19: Size of train, test, and validation splits, per subtype

| **Subtype** | **Train** | **Validation** | **Test** |
| --- | --- | --- | --- |
| **BLCA** | 291 | 45 | 46 |
| **BRCA** | 581 | 90 | 90 |
| **HNSC** | 338 | 52 | 53 |
| **KIRC** | 226 | 35 | 36 |
| **LIHC** | 274 | 42 | 43 |
| **LUAD** | 318 | 49 | 50 |
| **PAAD** | 134 | 21 | 21 |
| **SKCM** | 330 | 51 | 51 |

S20: Selected hyperparameters for trained models

| **Model** | **Learning Rate** | **Epochs** | **Batch Size** | **Weight Decay** |
| --- | --- | --- | --- | --- |
| Uni-Self-Omics VAE | 0.008 | 500 | 32 | 1e-4 |
| Uni-Self-Omics Survival Model | 0.0002 | 40 | 32 | 1e-4 |
| Uni-Self-WSI | 0.0001 | 40 | 4*4 | 1e-4 |
| Uni-Cross-Omics | 0.0002 | 40 | 32 | 1e-4 |
| Uni-Cross-WSI | 0.0001 | 40 | 4*4 | 1e-4 |
| Multi-Self | 0.0001 | 40 | 3*8 | 1e-4 |
| Multi-Cross | 0.0001 | 40 | 3*8 | 1e-4 |
| Uni-Transfer-Omics | 0.0002 | 40 | 32 | 1e-4 |
| Uni-Transfer-WSI | 0.0001 | 40 | 4*4 | 1e-4 |
| Multi-Transfer | 0.0001 | 10 | 3*8 | 1e-4 |
| Multi-Transfer (finetuning) | 0.0001 | 40 | 3*8 | 1e-4 |
